# Retrospective observational study of first-year developmental outcomes following congenital microcephaly

**DOI:** 10.1038/s41372-026-02650-9

**Published:** 2026-04-15

**Authors:** Morgan Chadwick, Kunal Koka, Allen Kunselman, Tammy E. Corr

**Affiliations:** 1 Penn State College of Medicine, Hershey, PA USA; 2 Penn State College of Medicine, Department of Public Health Sciences, Hershey, PA USA; 3 Penn State College of Medicine, Department of Pediatrics, Division of Neonatal-Perinatal Medicine, Hershey, PA USA; 4 Penn State Milton S. Hershey Medical Center Children’s Hospital, Hershey, PA USA

**Keywords:** Neurological manifestations, Neurodevelopmental disorders

## Abstract

**Objective:**

Determine the association between persistent versus resolved microcephaly and motor developmental outcomes in infants with congenital microcephaly.

**Study design:**

Bayley Scales of Infant and Toddler Development^®^ were collected for all children with congenital microcephaly seen in a NICU Follow-Up Clinic 2015–2024. A linear mixed model was fit to assess the relationship between microcephaly (resolved vs. persistent) with respect to Bayley motor composite scores in the first year.

**Results:**

Seventy-eight infants had head circumference measurements and Bayley motor composite scores at both visit 1 and visit 2. Infants with persistent microcephaly at 4-6 months had mean motor scores 12.07 (95% CI [-22.94, -1.19], *p* = 0.02) points lower than peers with resolved microcephaly. By 9–12 months, these differences were no longer significant.

**Conclusion:**

Persistent microcephaly was associated with lower motor development scores at 4-6 months compared to children with resolved microcephaly. This simple, anthropometric measurement may be useful in predicting early motor development.

## Introduction

Head circumference is a vital measurement useful for monitoring the growth and development of neonates, and it serves as a low-cost and effective tool for identifying infants at risk for potential neurodevelopmental disorders [1]. Within the general population, larger head circumference has been associated with increased intelligence, academic performance, and lifetime occupational prestige, establishing early head circumference as a potential predictor for later success. However, for preterm, very low birth weight, and small for gestational age infants, diagnoses that frequently lead to a neonatal intensive care unit (NICU) admission, the relationship between head circumference and neurodevelopmental outcomes remains largely unclear. For instance, some studies have shown congenital microcephaly, defined as head circumference <10^th^ percentile at birth, to be associated with poor motor and cognitive development [2–4]. However, other studies have concluded that head circumference at birth is not associated with motor and cognitive outcomes [5–7].

Postnatal catch-up head growth has also been shown to be associated with developmental outcomes. For example, in neonates with microcephaly at birth, postnatal head growth and catch-up to normal circumference percentiles has been correlated with improved developmental outcomes [2, 4, 6–8]. In contrast, a large retrospective cohort study of 4530 preterm infants found no association between postnatal head growth and developmental outcomes, when adjusted for comorbidities [9]. Thus, a complex, ambiguous interplay exists between head circumference at birth, postnatal growth, gestational age, and developmental outcomes.

Previous analyses have focused on specific populations, such as extremely preterm, very low-birth weight, or small for gestational age patients when determining the effect of congenital microcephaly and postnatal catch-up head circumference growth on developmental outcomes. However, less is understood about newborns who were born at term with normal growth parameters except for microcephaly, those with mixed diagnoses (e.g. preterm and meningitis), and those who are affected by moderate or late prematurity. Therefore, this study aimed to determine the effect of congenital microcephaly and postnatal catch-up growth on motor development of infants across gestational ages and clinic-qualifying diagnoses utilizing the updated Bayley Scales of Infant and Toddler Development^®^ (BSID), third and fourth editions.

## Methods

This single center, retrospective chart review was performed by retrieving electronic medical records (EMR) of patients who were cared for in the Penn State Milton S. Hershey Medical Center Neonatal Intensive Care Unit (NICU) from January 2015 to July 2024. Penn State Medical Center is a tertiary care, academic institution with approximately 2300 deliveries per year, an active Maternal-Fetal Medicine program, and a 56-bed level IV NICU with roughly 800 admissions per year, including nearly 250 neonates transported in from outside hospitals.

### Patient population

The EMR system was queried for eligible records of NICU patients who were seen in the Penn State NICU Neurodevelopmental Follow-Up Clinic. Inclusion criteria encompassed infants with birth head circumference <10th percentile as calculated by the 2013 Fenton Growth Chart for Preterm Infants, a NICU stay at Penn State Children’s Hospital during the period of 2015–2024, a clinic-qualifying diagnosis such as prematurity or hypoxic injury (isolated congenital microcephaly was not an eligible criterion for neurodevelopmental follow-up), and BSID III/IV scores at both 4–6 months and 9–12 months corrected age. Exclusion criteria included birth head circumference ≥10th percentile, infants without BSID III/IV scores at 4–6 months and 9–12 months, or infants with a known genetic or chromosomal anomaly or syndrome that is associated with abnormal neurodevelopmental outcomes.

### Data collection

If the neonate met inclusion criteria, further clinical data were extracted manually by the authors (MC, KK, TEC). No specific data collection tool was used in the study. Relevant maternal and neonatal data related to pregnancy health and the postnatal NICU course were documented (see Table 1). Growth data, including weight, length, and head circumference as well as growth percentiles were collected at birth and hospital discharge as well as at each clinic visit. Birth measurements, including head circumference, are obtained upon admission to the NICU or, if the infant is transferred from an outside hospital, collected from transfer paperwork. The standard procedure for obtaining a head circumference measurement in our NICU involves a trained nurse taking a single measurement using a non-stretchable, paper tape measurer, placing it above the supine infant’s brow line, and pulling it around the most prominent part of the occiput. The measurement is rounded to the nearest 0.1 cm and recorded. The same method is used in the NICU Neurodevelopmental Follow-Up Clinic, though an older infant may have a measurement obtained in a sitting position. Growth percentiles were plotted on the 2013 Fenton Growth Chart for all infants postnatally, transitioning to World Health Organization (WHO) Child Growth Standards during the NICU admission if admitted beyond 50 weeks corrected gestational age. All outpatient, NICU Clinic measurements were plotted on the WHO Growth Curve.

**Table 1:** Clinical characteristics.

Infant Characteristics	Total (*N* = 78)
**Sex**, n (%)	
Male	38 (48.7%)
Female	40 (51.3%)
**Gestational Age (weeks)**	
Mean (SD)	31.6 (4.3)
Median (IQR)	32.0 (28.3, 34.1)
Range	23.1, 41.1
**Birth Year**, n (%)	
2015	0 (0.0%)
2016	4 (5.1%)
2017	0 (0.0)
2018	0 (0.0)
2019	5 (6.4%)
2020	8 (10.3%)
2021	16 (20.5%)
2022	26 (33.3%)
2023	19 (24.4%)
**Birth Head Circumference Percentile (Fenton)**	
Mean (SD)	4.7 (2.7)
Median (IQR)	5.0 (3.0, 7.0)
Range	0.0, 9.0
**Birth Length Percentile (Fenton)**	
Mean (SD)	15.8 (15.5)
Median (IQR)	9.0 (4.0, 24.0)
Range	0.0, 70.0
**Birth Weight Percentile (Fenton)**	
Mean (SD)	14.3 (15.0)
Median (IQR)	9.0 (5.0, 18.0)
Range	0.0, 72.0
**Inborn Status**, n (%)	64 (82.1%)
Inborn	14 (17.9%)
Outborn	
**5-Min Apgar Score**, n (%)	
<6	9 (11.5%)
≥6	68 (87.2%)
Missing	1 (1.3%)
**Intraventricular Hemorrhage**, n (%)	
None	67 (85.9%)
Grade I	8 (10.3%)
Grade II	1 (1.3%)
Grade III	1 (1.3%)
Grade IV	1 (1.3%)
**Periventricular Leukomalacia**, n (%)	
No	76 (97.4%)
Yes	2 (2.6%)
**Late-onset Sepsis (>72 h)**, n (%)	
No	75 (96.2%)
Yes	3 (3.8%)
**Bronchopulmonary Dysplasia**	
No	53 (67.9%)
Yes	25 (32.1%)
**Human Milk at Discharge**, n (%)	
No	21 (26.9%)
Yes	57 (73.1%)
**NICU Length of Stay (days)**	
Mean (SD)	64.5 (64.3)
Median (IQR)	41.5 (23.0, 85.0)
Range	3.0, 464.0
**Clinic Qualifying Diagnosis**, n (%)	
Prematurity (< 35 weeks)	66 (84.6%)
Neonatal Abstinence Syndrome	4 (5.1%)
Very Low Birthweight Infant	2 (2.6%)
Hypoxic Ischemic Encephalopathy	1 (1.3%)
Seizures	1 (1.3%)
Congenital Infection	1 (1.3%)
Meningitis	1 (1.3%)
Other	2 (2.6%)
**Maternal Characteristics**	
**Antenatal Steroids**, n (%)	
None	23 (29.5%)
At least 1 dose prior to delivery	55 (70.5%)
**Chorioamnionitis**, n (%)	
No	74 (94.9%)
Yes	3 (3.8%)
Missing	1 (1.3%)
**Preeclampsia**, n (%)	
No	44 (56.4%)
Yes	34 (43.6%)
**Mode of Delivery**, n (%)	
C-section	63 (80.8%)
Vaginal	14 (17.9%)
Unknown	1 (1.3%)
**Visit Characteristics**	
**Bayley’s Testing Version at Visit 1**, n (%)	
III	11 (14.1%)
IV	67 (85.9%)
**Bayley’s Testing Version at Visit 2**, n (%)	
III	6 (7.7%)
IV	72 (92.3%)

Study data were managed using Research Electronic Data Capture (REDCap) tools hosted at Penn State Health Milton S. Hershey Medical Center and Penn State College of Medicine. This study was approved by Penn State’s Institutional Review Board, STUDY00025368.

### Statistical analysis

For the analysis, microcephaly status was classified as resolved (head circumference >≥10th percentile) or persistent (head circumference remaining below the 10th percentile) at each NICU Clinic visit in the first year of life. While there was no control group in this retrospective study as all eligible infants were classified as having microcephaly at birth, infants classified as having newly resolved microcephaly at 4–6 months (i.e., visit 1) served as an adequate comparator group. A linear mixed model [10]. which accounted for the repeated measures over visits (i.e., over time), was fit to assess the direction and strength of the association between microcephaly status and BSID motor composite scores. The model consisted of a random subject effect and fixed effects for microcephaly status, visit, the interaction of microcephaly status and visit, and the covariates of infant’s sex, gestational age, year of birth, version of Bayley’s test (BSID III or IV), preeclampsia during pregnancy, presence of sepsis or meningitis, and presence of periventricular leukomalacia (PVL), grade III-IV intraventricular hemorrhage (IVH), or ventriculoperitoneal (VP) shunt. The variance-covariance structure was compound symmetry for this mixed model with repeated BSID motor composite scores at two time points.

Adherence to linear mixed model assumptions were verified by assessing residual versus predicted value plots for homoscedasticity and model fit, quantile-quantile plots for normality, testing for homogeneity of variances across the microcephaly status classifications, adding polynomials to the model and examining plots of residuals versus continuous independent variables to assess linearity, and having an intraclass correlation coefficient (ICC) of 0.47 from the linear mixed model verifying the need for a random effect. Contrasts were constructed from the linear mixed model to compare microcephaly status over time adjusting for the covariates.

To control the familywise error rate, these post-hoc comparisons were corrected for multiple hypothesis testing using simulation-based methods [11]. Additionally, linear mixed models were used to assess the association of each individual covariate (e.g., infant’s sex) with BSID motor composite scores. Significance was defined as *p* < 0.05. All hypothesis tests were two-sided and statistical analyses were performed using SAS software, version 9.4 (SAS Institute Inc., Cary, NC).

## Results

After applying inclusion and exclusion criteria, 78 neonates were eligible to participate in the study. All 78 infants had head circumference measurements and BSID motor composite scores at both visit 1 (4–6 months) and visit 2 (9–12 months). Furthermore, there were no missing data for any of the covariates considered (e.g., infant’s sex, gestational age, etc.) for these 78 infants. The sample was evenly split between males and females. Gestational age at birth ranged from 23–41 weeks. The majority of infants (88.5%) were born during the last 4 years of data collection (2020–2023), a byproduct of expanded NICU Clinic eligibility criteria and a move to a new unit in 2020 that increased the number of NICU beds by 33%. Most children were symmetrically small with median length and weight percentiles of 9.0% (interquartile range [IQR] 4.0, 24.0) and 9.0% (IQR 5.0, 18.0), respectively. Complications such as severe grade (III-IV) IVH and PVL occurred in a minority of the population (2.6% and 2.6%, respectively) while late-onset sepsis (3.8%) and bronchopulmonary dysplasia (32.1%) occurred more frequently. The median length of stay was 42 days, and the majority (73.1%) of the infants were discharged feeding human milk. Infants qualified for NICU Follow-Up Clinic for a variety of reasons, though prematurity was the most frequent reason (84.6%). Most infants were evaluated using BSID version IV (Table 1).

Of the 78 infants with congenital microcephaly seen at 4-6 months corrected age (NICU Clinic visit 1), 22 (28.2%) had persistent microcephaly, while 56 (71.8%) infants had resolution of their microcephaly (Table 2). By the second NICU Clinic visit at 9-12 months corrected age, 5 additional infants had resolution of their microcephaly, 17 maintained microcephaly, 51 maintained normocephaly, and 5 had recurrence of their microcephaly (Table 2, Supplementary Fig. 1).

**Table 2:** Bayley motor composite scores.

Microcephaly Status	Visit	Number	Mean (SD)	Median (IQR)	Range
Persistent	1	22	81.0 (16.3)	83.5 (67.0, 94.0)	52.0, 107.0
	2	17	84.9 (19.4)	88.0 (76.0, 97.0)	49.0, 112.0
Newly Resolved	1	56	94.3 (15.2)	97.0 (85.0, 107.0)	58.0, 118.0
	2	5	81.6 (22.6)	88.0 (70.0, 94.0)	49.0, 107.0
Persistently Resolved	2	51	90.1 (17.9)	91.0 (82.0, 103.0)	46.0, 118.0
Recurrence	2	5	91.4 (17.6)	88.0 (85.0, 107.0)	67.0, 110.0

**Fig. 1:**
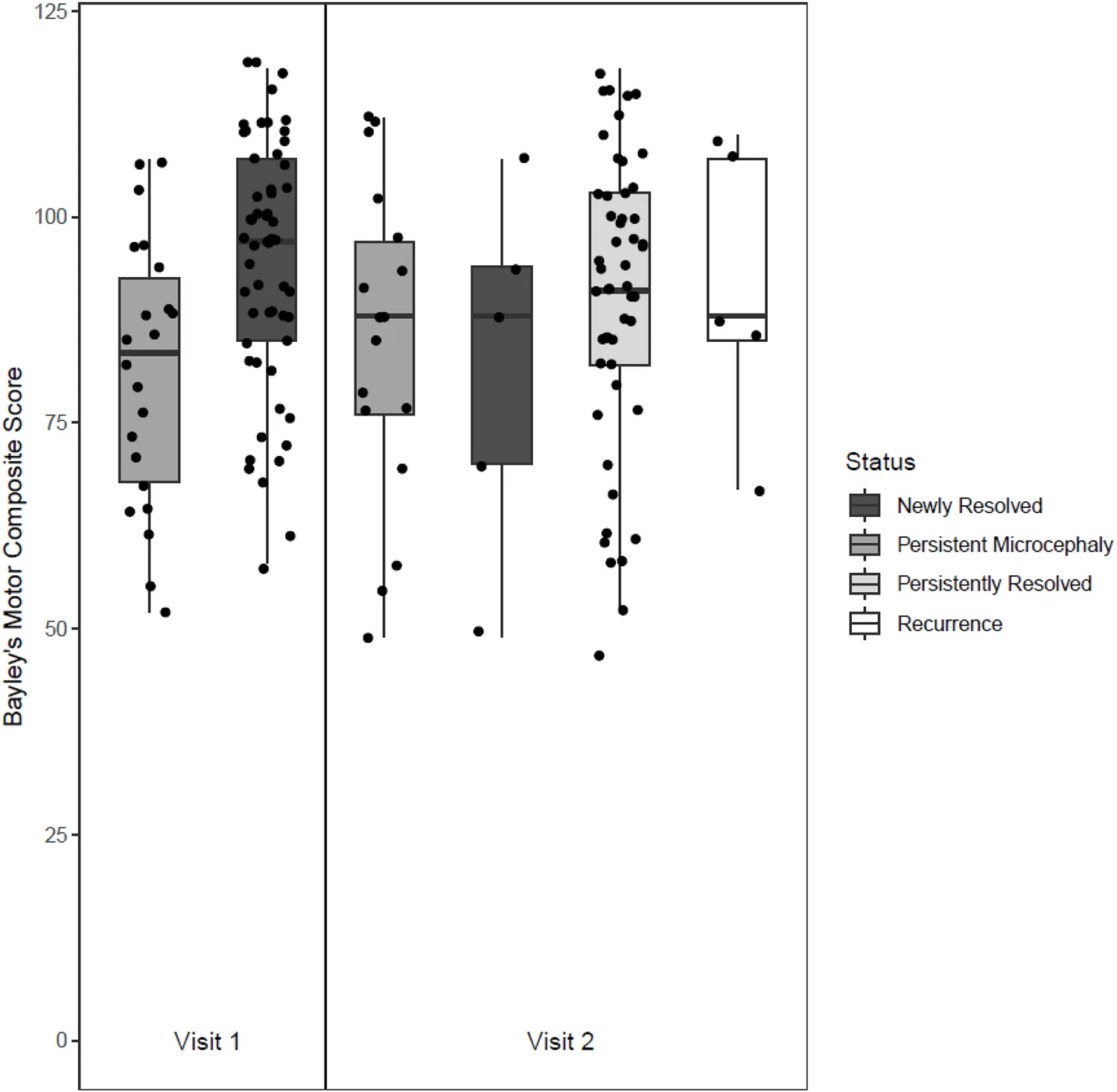
Bayley’s Scales of Infant and Toddler Development Composite Motor Scores. Visit 1 occurs at 4-6 months corrected age. Visit 2 occurs at 9–12 months corrected age.

For those infants with persistent microcephaly at NICU Clinic visit one, the median BSID motor composite score was 83.5 (IQR 67.0, 94.0), while the median score of infants with resolved microcephaly at this first visit was 97.0 (IQR 85.0–107.0) (Table 2). At visit two, those with persistence or recurrence of their microcephaly had median motor composite scores of 88.0 (IQR 76.0, 97.0) and 88.0 (IQR 85.0, 107.0), respectively, while those with newly resolved or persistently resolved microcephaly had median motor composite scores of 88.0 (IQR 70.0, 94.0) and 91.0 (IQR 82.0, 103.0), respectively (Table 2, Fig. 1). Thirteen infants were seen at the 18-month follow-up visit as well, where language and cognition were evaluated in addition to motor skills. However, they were not included in the study analysis due to the small cohort with some infants also missing data at earlier clinic visits due to a missed or canceled visit.

**Fig. 2:**
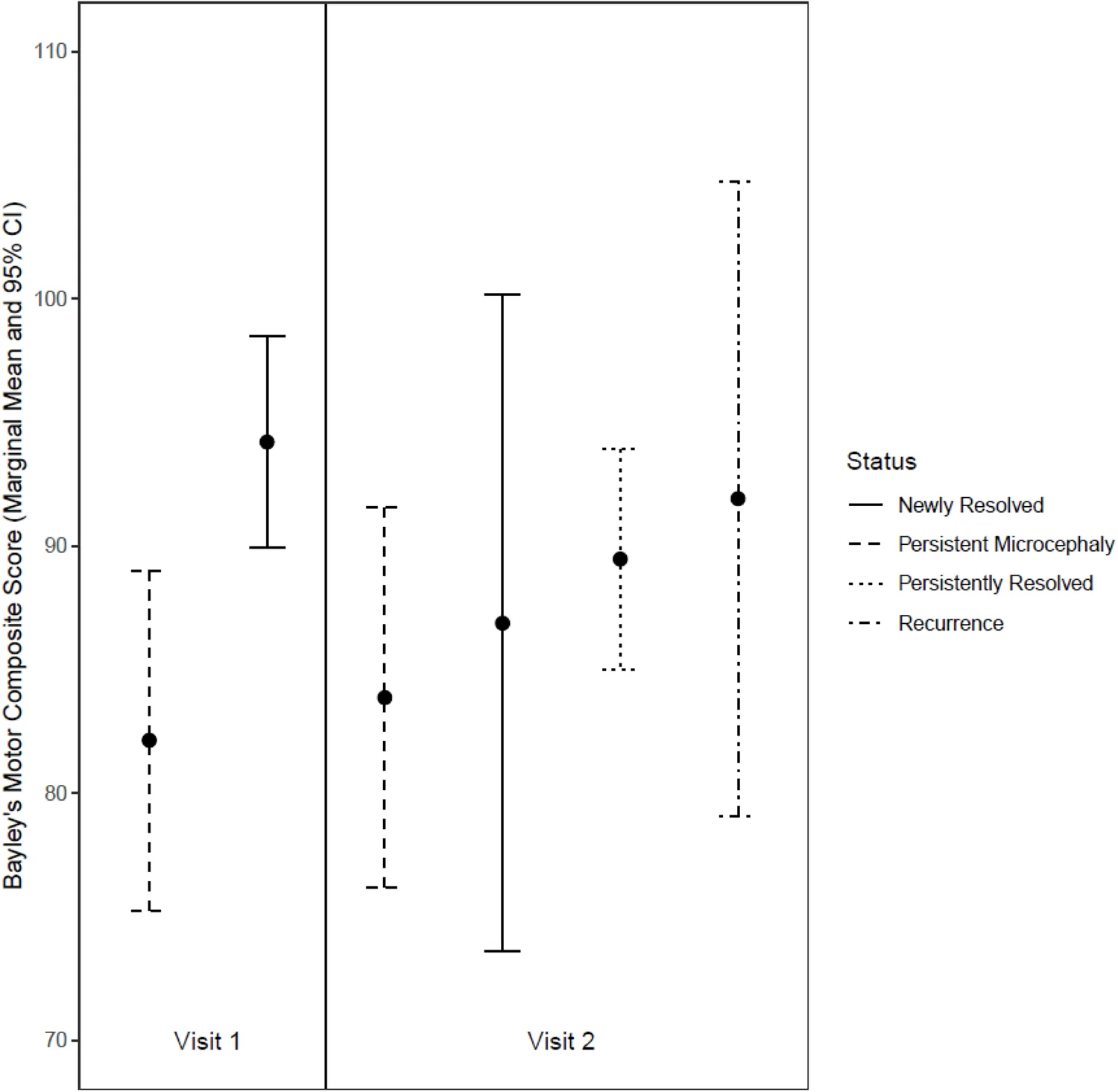
Marginal Means of Bayley’s Scales of Infant and Toddler Development Composite Motor Scores. Visit 1 occurs at 4-6 months corrected age. Visit 2 occurs at 9-12 months corrected age. Scores are reported as marginal means and 95% confidence intervals.

Utilizing a linear mixed model and adjusting for covariates, tests of fixed effects resulted in marginal means of BSID motor composite scores were calculated and adjusted for multiple comparisons. Infants with persistent microcephaly at their 4-6 month NICU Clinic visit had mean BSID motor scores 12.07 (95% confidence interval [95% CI] -22.94, -1.19; *p* = 0.02) points lower than their peers with resolved microcephaly (Table 3 and Fig. 2). However, by the 9–12 month visit, these differences in scores between infants with persistent and newly-resolved microcephaly were no longer significant (-3.01, 95% CI [-22.52, 16.50]; *p* = 0.99). Additional comparisons of marginal means of BSID motor composite scores were made at the 9–12 month visit, including between persistent microcephaly and persistently-resolved microcephaly, persistent microcephaly and recurrent microcephaly, newly-resolved and persistently-resolved microcephaly, newly-resolved and recurrent microcephaly, and persistently-resolved and recurrent microcephaly with no significant differences found between any of the groups (Table 3, Fig. 2). Infant profile plots of BSID scores between visit 1 and 2 for infants who changed microcephaly categories between visits did not reveal any specific or informative patterns (Supplementary Fig. 1).

**Table 3:** Differences in Marginal Means of Bayley Motor Composite Scores Adjusted for Multiple Comparisons.

Microcephaly Status and Visit Number	Difference in Marginal Means (95% CI)	*p*-value
Persistent Microcephaly Visit 1 vs. Newly Resolved Visit 1	-12.07 (-22.94, -1.19)	0.02
Persistent Microcephaly Visit 2 vs. Newly Resolved Visit 2	-3.01 (-22.52, 16.50)	0.99
Persistent Microcephaly Visit 2 vs. Persistently Resolved Visit 2	-5.61 (-17.56, 6.35)	0.67
Newly Resolved Visit 2 vs. Persistently Resolved Visit 2	-2.60 (-21.22, 16.03)	0.99
Persistently Resolved Visit 2 vs. Recurrent Microcephaly Visit 2	-2.44 (-20.26, 15.37)	0.99
Newly Resolved Visit 2 vs. Recurrent Microcephaly Visit 2	-5.04 (-29.63, 19.55)	0.98
Persistent Microcephaly Visit 2 vs. Recurrent Microcephaly Visit 2	-8.05 (-27.85, 11.75)	0.77

Additionally, each covariate was independently assessed for association with the BSID motor composite scores (Supplementary Table 1). BSID motor composite scores were higher in infants as gestational age and year of birth increased, as well as in infants without a diagnosis of late onset sepsis or meningitis and in infants evaluated using the Bayley’s IV testing version. There was no significant association between BSID motor scores and infant’s sex, maternal presence of preeclampsia, or infant presence of grade III or IV IVH, VP shunt, or PVL.

Finally, to limit bias in the analysis, two sensitivity analyses were conducted. The first analysis evaluated only premature infants with a gestational age <35 weeks at birth (*n* = 66) and found that results were similar to the full dataset of infants (*n* = 78). Specifically, the marginal mean difference in Bayley’s motor composite scores for persistent microcephaly vs. newly resolved microcephaly at visit 1 in premature infants was -12.20 (95% CI: –24.40 to –0.01; *p*-value = 0.05) compared to the full cohort of infants (term and preterm) where persistent microcephaly was associated with a marginal mean difference in Bayley motor scores of -12.07 (95% CI: –22.94 to –1.19; *p*-value = 0.02). Other comparisons of marginal mean differences in Bayley scores for the preterm subanalysis were not statistically significant.

Because the Fenton growth curves were designed for premature infants but are used on all neonates born at our institution, a second sensitivity analysis removed term infants whose dichotomous microcephaly classification (head circumference <10^th^ percentile) differed on the Fenton and World Health Organization (WHO) growth scales (*n* = 3) and focused on infants with a gestational age ≥ 38 weeks whose classification of microcephaly agreed on both scales (total *n* = 75). When comparing persistent microcephaly vs. newly resolved microcephaly at visit 1 for the subset, the marginal mean differences in Bayley’s motor composite scores was -12.40 (95% CI: –23.64 to –1.16; *p*-value = 0.02) compared to the full set of infants (*n* = 78) where the marginal mean difference was -12.07 (95% CI: –22.94 to –1.19; p-value = 0.02). Other comparisons remained statistically insignificant.

## Discussion

The results of this study highlight a significant association between the persistence or resolution of congenital microcephaly and developmental motor scores at a 4-6 month NICU Neurodevelopmental Follow-Up Clinic visit. Neonates whose microcephaly was persistent at the first NICU Clinic appointment demonstrated significantly lower mean BSID composite motor scores compared to those with resolution of microcephaly by their 4-6 month visit. However, by the 9-12 month visit, there were no statistically-significant differences in mean motor scores between infants with persistent microcephaly and those with newly-resolved or persistently-resolved microcephaly. These findings suggest that early normalization of head circumference may be a favorable prognostic indicator for early motor development but may be less useful in predicting outcomes as an infant’s first birthday approaches.

These results contribute to a growing body of evidence that links persistent microcephaly with poorer developmental outcomes [3–5]. Poor performance on early developmental testing is associated with a number of adverse outcomes into later childhood and adulthood, including worse academic achievement and educational attainment, lower income, and unemployment [12–14].

In children with congenital microcephaly, monitoring the progression of their head growth may be a simple and effective way to determine which children remain most at risk for these future, potential adverse outcomes. For example, infants with catch-up head growth and resolution of their microcephaly in the early months following birth may be at less risk for poor development, while those infants with persistent microcephaly may require closer monitoring and early referral to therapy services.

The results of this study suggest that for those children without catch-up growth into normal head circumference percentiles by 6 months of age, engagement with early intervention services should be prioritized. Early intervention in the infant and toddler years has been associated with improvements in neurodevelopmental, neurobehavioral, and cognitive outcomes in childhood [15–18]. In fact, it is possible that the absence of significant differences in scores observed at 9–12 months in our own data may reflect the impact of Early Intervention therapy services on those children with ongoing microcephaly and poor BSID motor scores. Indeed, per our NICU Clinic protocol, any child who performs poorly on their developmental assessment is referred to county Early Intervention programs for engagement in therapy services, is sent back to their Early Intervention therapist for an increase or modification of services, and/or is referred to outpatient physical, occupational, or speech therapy for additional services, all of which may potentially mitigate delays by the next follow-up visit.

Despite its contributions, this study is limited by a relatively small sample size, with only 78 microcephalic neonates eligible for study inclusion over an eight-year period. The attrition rate was significant with approximately 25% of nearly 200 qualifying patients seen at their first clinic visit attending their final visit at 18-24 months of age, forcing a restriction of outcome evaluation to the first year of life and first two clinic visits. The COVID-19 pandemic further constrained data collection, as many families missed follow-up appointments or staffing shortages limited available appointments, and some records lacked BSID score documentation. In addition, though we set out to evaluate the developmental trajectory of preterm and term infants with a variety of clinic-qualifying diagnoses, a full 85% of our sample was referred to our NICU Neurodevelopmental Follow-Up Clinic for prematurity, making application of our results somewhat limited. We also could not readily account for whether an infant received Early Intervention therapy services, and this information would have added to the interpretation of results. Measurement inconsistencies posed another challenge. In some cases, microcephaly appeared to resolve at the 4–6 month visit but reappeared at 9–12 months, raising questions of errors in measurement. Additionally, this study was unable to assess the relationship between microcephaly and language or cognitive testing, as there were few neonates who attended the 18-month NICU Developmental Clinic follow up appointments.

Nonetheless, the findings of this study offer valuable insight into the developmental trajectories of microcephalic neonates and reinforce the importance of early head circumference monitoring.

These findings may also inform NICU Clinic follow-up protocols, such as tailoring visit frequency or intervention plans for infants with persistent microcephaly. Overall, the data contribute to the existing literature around the association of head circumference and head growth with neurodevelopmental outcomes in childhood. However, there is still much more work to be conducted for a better understanding of this topic. Avenues for future research include similar, prospective studies with larger sample sizes, noting which infants are referred to Early Intervention or intensive therapy services after their initial visits, and the inclusion of multicenter cohorts. Additionally, long-term follow-up into childhood and beyond would better characterize the lasting impact of persistent versus resolved microcephaly on neurodevelopment.

## Conclusion

This study supports the clinical utility of head circumference as an early marker of motor developmental risk in NICU graduates. This simple, anthropometric measurement may be a useful predictive tool for early motor performance and may aid in parental counseling and child engagement in Early Intervention services, potentially improving development over time. Continued research in this area will enhance understanding and guide interventions aimed at improving outcomes for this vulnerable population.

## Supplementary information

Supplemental Material

## Data Availability

Deidentified data sets are available upon request of the authors.

## References

[1] Harris SR. Measuring head circumference: Update on infant microcephaly. Can Fam Phys. 2015;61:680–4. PMC4541430 26505062

[2] Hong YM, Cho DH, Kim JK. Developmental outcomes of very low birth weight infants with catch-up head growth: a nationwide cohort study. BMC Pediatr. 2023;23:392. 37553623 10.1186/s12887-023-04135-6 PMC10408187

[3] Raz S, Newman JB, DeBastos AK, Peters BN, Batton DG. Postnatal growth and neuropsychological performance in preterm-birth preschoolers. Neuropsychology. 2014;28:188–201. 24364394 10.1037/neu0000038

[4] Selvanathan T, Guo T, Kwan E, Chau V, Brant R, Synnes AR, et al. Head circumference, total cerebral volume and neurodevelopment in preterm neonates. Arch Dis Child Fetal Neonatal Ed. 2022;107:181–7. 34261769 10.1136/archdischild-2020-321397

[5] Cooke RW. Foulder-Hughes L. Growth impairment in the very preterm and cognitive and motor performance at 7 years. Arch Disease Child. 2003;88:482–7. 12765911 10.1136/adc.88.6.482 PMC1763118

[6] Kan E, Roberts G, Anderson PJ, Doyle LW. The association of growth impairment with neurodevelopmental outcome at eight years of age in very preterm children. Early Hum Dev. 2008;84:409–16. 18096332 10.1016/j.earlhumdev.2007.11.002

[7] Kuban KC, Allred EN, O’Shea TM, Paneth N, Westra S, Miller C, et al. Developmental correlates of head circumference at birth and two years in a cohort of extremely low gestational age newborns. J Pediatr. 2009;155:344–9.e1-3. 19555967 10.1016/j.jpeds.2009.04.002 PMC2803763

[8] Ghods E, Kreissl A, Brandstetter S, Fuiko R, Widhalm K. Head circumference catch-up growth among preterm very low birth weight infants: effect on neurodevelopmental outcome. J Perinat Med. 2011;39:579–86. 21740330 10.1515/jpm.2011.049

[9] Bando N, Fenton TR, Yang J, Ly L, Luu TM, Unger S, et al. Association of postnatal growth changes and neurodevelopmental outcomes in preterm neonates of <29 weeks’ gestation. J Pediatr. 2023;256:63–69.e2. 36509160 10.1016/j.jpeds.2022.11.039

[10] Brown H, Prescott R Applied mixed models in medicine (2^nd^ ed.). Wiley, 2006.

[11] Edwards D, Berry JJ. The efficiency of simulation-based multiple comparisons. Biometrics. 1987;43:913–28. 3427176

[12] Schmitt SA, Duncan RJ, Paes TM, Vandell DL. Identifying the onset and magnitude of prediction from early cognitive skills to adult socioeconomic outcomes. Child Dev. 2025;0:1–15. 10.1111/cdev.70006 PMC12598447 40755036

[13] Burchinal M, Vandell DL. School Entry Skills and Young Adult Outcomes. Early Child Res Q. 2025;72:1–12. 40027937 10.1016/j.ecresq.2025.01.004 PMC11870661

[14] Williams KE, Berthelsen D, Laurens KR. Academic resilience from school entry to third grade: child, parenting, and school factors associated with closing competency gaps. PLoS ONE. 2022;17:e0277551. 36449482 10.1371/journal.pone.0277551 PMC9710847

[15] Bernabe-Zuñiga JE, Rodriguez-Lucenilla MI, Alias-Castillo AJ, Rueda-Ruzafa L, Roman P, Del Mar Sanchez-Joya M. Early interventions with parental participation and their implications on the neurodevelopment of premature children: a systematic review and meta-analysis. Eur Child Adolesc Psych. 2025;34:853–65. 10.1007/s00787-024-02528-1 39028424

[16] Reynolds AJ, Ou SR, Mondi CF, Hayakawa M. Processes of early childhood interventions to adult well-being. Child Dev. 2017;88:378–87. 28195326 10.1111/cdev.12733 PMC5453306

[17] Khurana S, Kane AE, Brown SE, Tarver T, Dusing SC. Effect of neonatal therapy on the motor, cognitive, and behavioural development of infants born preterm: a systematic review. Dev Med Child Neurol. 2020;62:684–92. 32077096 10.1111/dmcn.14485 PMC7920849

[18] Spittle A, Treyvaud K. The role of early developmental intervention to influence neurobehavioral outcomes of children born preterm. Semin Perinatol. 2016;40:542–8. 27817913 10.1053/j.semperi.2016.09.006

