# Supplementary material for: Retrospective observational study of first-year developmental outcomes following congenital microcephaly
Source: J Perinatol. 2026 Apr 15;46(9):1794–800. doi: 10.1038/s41372-026-02650-9 (PMC13588750; doi:10.1038/s41372-026-02650-9)
Supplement: Supplementary file 1 [file 41372_2026_2650_MOESM1_ESM.pdf]

Supplementary Material:

Supplemental Figure 1. Infant Profile Plots of Bayley's Motor Composite Scores. Visit 1 occurred at 4-6 months corrected age. Visit 2 occurred at 9-12 months corrected age.

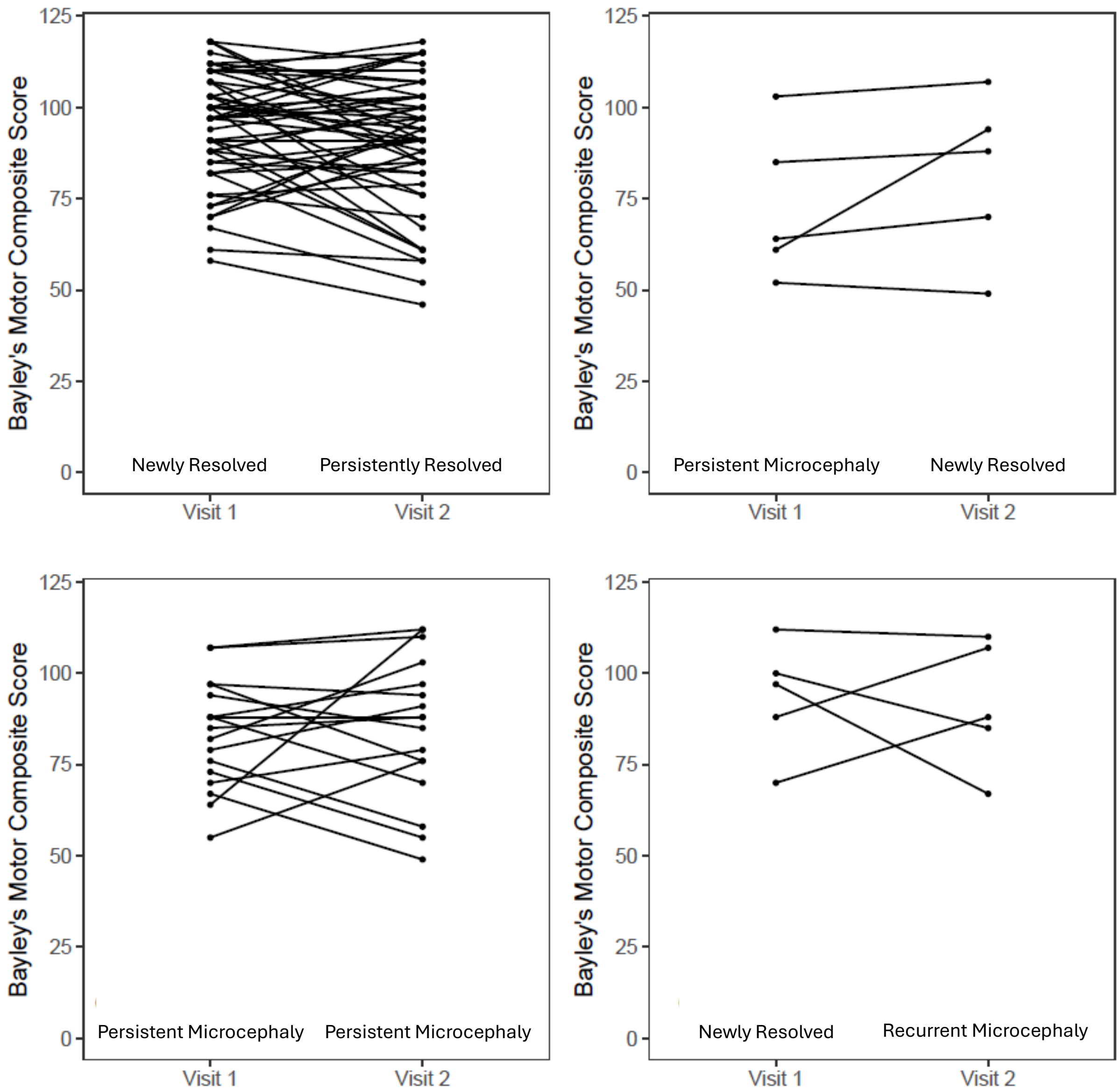

Supplemental Table 1. Independent Covariate Associations with Bayley's Motor Composite Scores

Covariate	Bayley's Score Estimate (95% CI)	*p*-value
Sex		0.13
Male	92.25 (87.33, 97.17)	
Female	86.91 (82.11, 91.71)	
Bayley Test Method		0.02
III	79.28 (69.94, 88.62)	
IV	90.89 (87.40, 94.37)	
IVH[1] Grades III-IV, VP[2] Shunt, and/or PVL[3]		0.28
No	89.89 (86.36, 93.43)	
Yes	80.00 (62.34, 97.66)	
Late Onset Sepsis or Meningitis		0.01
No	90.51 (87.07, 93.95)	
Yes	71.13 (56.33, 85.92)	
Preeclampsia		0.50
No	88.47 (83.83, 93.10)	
Yes	90.87 (85.60, 96.14)	
Gestational Age (increase per week)	1.06 (0.28, 1.84)	0.01
Birth Year (increase per year)	2.85 (0.89, 4.82)	0.01

[1]IVH=intraventricular hemorrhage, [2]VP=ventriculoperitoneal, [3]PVL=periventricular leukomalacia
